# Fast environmental sound classification based on resource adaptive convolutional neural network

**DOI:** 10.1038/s41598-022-10382-x

**Published:** 2022-04-22

**Authors:** Zheng Fang, Bo Yin, Zehua Du, Xianqing Huang

**Affiliations:** 1 College of Information Science and Engineering, Ocean University of China, Qingdao, China; 2 Pilot National Laboratory for Marine Science and Technology, Qingdao, China

**Keywords:** Computer science, Acoustics

## Abstract

Recently, with the construction of smart city, the research on environmental sound classification (ESC) has attracted the attention of academia and industry. The development of convolutional neural network (CNN) makes the accuracy of ESC reach a higher level, but the accuracy improvement brought by CNN is often accompanied by the deepening of network layers, which leads to the rapid growth of parameters and floating-point operations (FLOPs). Therefore, it is difficult to transplant CNN model to embedded devices, and the classification speed is also difficult to accept. In order to reduce the hardware requirements of running CNN and improve the speed of ESC, this paper proposes a resource adaptive convolutional neural network (RACNN). RACNN uses a novel resource adaptive convolutional (RAC) module, which can generate the same number of feature maps as conventional convolution operations more cheaply, and extract the time and frequency features of audio efficiently. The RAC block based on the RAC module is designed to build the lightweight RACNN model, and the RAC module can also be used to upgrade the existing CNN model. Experiments based on public datasets show that RACNN achieves higher performance than the state-of-the-art methods with lower computational complexity.

## Introduction

With the rise of deep learning technology, speech recognition technology has become more and more mature, and even surpasses the accuracy of artificial classification in the fields of human voice classification and music sound classification. However, as an important part of speech recognition, environmental sound classification still faces great challenges. ESC is widely used in smart home, scene analysis, machine hearing and other fields. Its goal is to accurately classify a class of detected sounds, such as car horn, engine idling, street music and so on. Due to the large non-stationary characteristics of environmental sound and the strong interference of environmental noise, it is difficult to classify.

**Figure 1:**
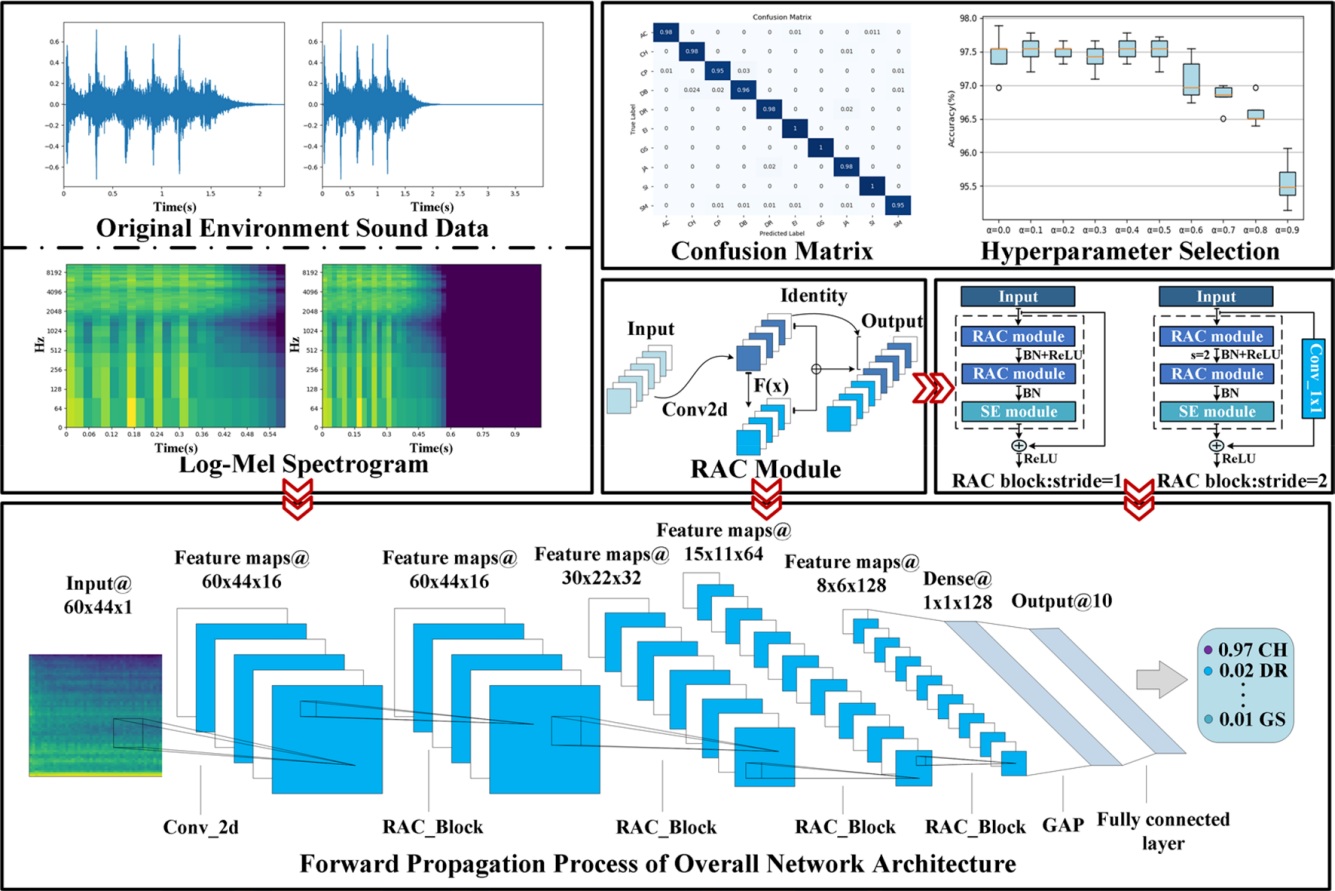
The specific process of ESC using RACNN. (Heat maps are drawn with the audio processing library Librosa 0.8.1. Detailed descriptions are available at https://librosa.org/doc/latest/index.html).

ESC mainly includes two steps: acoustic feature extraction and classifier. In order to extract acoustic features effectively, it is necessary to divide the sound signal into frames, and then extract features from each frame. Mel frequency cepstral coefficients (MFCC) and log-Mel spectrogram are two widely used features in ESC. In the early years, support vector machine (SVM)^1, 2^, Gaussian mixture model (GMM)^3^, extreme learning machine (ELM) and other machine learning algorithms were usually used to classify the extracted sound features. However, these traditional classifiers were designed to simulate small changes, which led to the lack of time and frequency invariance. In recent years, the method based on deep neural network (DNN) has been proved to be more effective in solving complex classification problems, and gradually replaced the traditional machine learning algorithm. Convolutional neural network, as one of the most commonly used architectures in deep learning, can learn in time and frequency simultaneously through convolution operation, which solves the limitations of traditional machine learning algorithm. At the same time, CNN can further extract deeper abstract features for classification on the basis of hand-made features. Although CNN has excellent performance, the improvement of its performance depends on a large number of parameters and FLOPs. The large number of parameters and calculations slow down the running speed of CNN, which makes it difficult to meet the requirements of real-time performance, and deploy to embedded devices which lack of storage and computing resources. Therefore, in order to reduce the operation cost of CNN and improve the classification speed of environmental sound, we propose RACNN, the core of which lies in a new convolution operation idea. We call it RAC module, which generates redundant feature maps in a relatively cheap way. Compared with the traditional convolution operation, it generates the same number of channels with lower storage and operation cost and gets more abundant feature information. On its basis, the channel domain attention mechanism and skip connection are fused to generate an efficient feature extraction block and RACNN is formed by simply stacking this block. The specific process of ESC using RACNN is shown in Fig. 1.

In summary, the main contributions of this paper are as follows:

- This paper proposes an efficient RAC module, which reduces the number of parameters and FLOPs of traditional convolution operation, and can trade-off between accuracy and efficiency according to the actual situation.
- Combining the shortcut and the channel domain attention mechanism, we build an efficient feature extraction block-RAC block, and build RACNN by stacking this block.
- Experiments based on public datasets show that RACNN achieves a trade-off between accuracy and efficiency.

The rest of this paper is organized as follows. “Related work ” section introduces the related work. “Method ” section introduces our RACNN and data preprocessing method. In “Experiment” section, the proposed method is verified by experiments. Finally, a summary of the whole paper is given.

## Related work

Deep learning has been widely used in various fields. In recent years, many scholars have introduced this technology into the field of ESC. In this chapter, we will introduce deep learning methods applied in ESC-related fields and mainstream research on CNN compression.

The earliest and most commonly used CNN model in the field of ESC is 2-D CNN. Piczak^4^ first proposed the use of 2-D CNN to learn Log-Mel spectrogram features, which has significantly improved ESC performance compared with traditional machine learning algorithms such as KNN and SVM. Chen et al.^5^ accurately identified the audio signal of the vehicle by fusing the LSTM unit into the convolutional neural network. Boddapati et al.^6^ uses AlexNet^7^ and GoogLeNet^8^ to classify the environmental sound features extracted from the spectrum. However, these CNN were used to classify the large image dataset-ImageNet at the earliest time. Therefore, these models are not fully suitable for the task of ESC, which is easy to cause overfitting, unable to give full play to CNN performance, cause redundancy of parameters and slow down the speed. Subsequently, many scholars began to study the influence of different spectrogram features on the final classification results. Tran et al.^9^ proposed SirenNet and combined the original audio waveforms, MFCC and Log-Mel as input for emergency vehicle detection based on sirens. Later, Su et al.^10^ used two combined features (MFCC-CST and LM-CST) to train CNN (MCNet and LMCNet), and then used Dempster-Shafer evidence theory (DS) to fuse CNN trained by different features to form TSCNN-DS model, which achieved 97.2% classification accuracy on UrbanSound8K dataset. Su et al.^11^ further analyzed the performance of ESC based on multi-aggregation acoustic features. Through a large number of experiments, the author found the best feature aggregation strategy among the feature combinations including MFCC, Log-Mel, Chroma, Spectral Contrast and Tonnetz to improve the accuracy of ESC. Finally, by fusing MFCC, Log-Mel, Spectral Contrast and Tonnetz, the accuracy of ESC-50 and UrbanSound8K is 85.6% and 93.4% respectively.

In addition to the most commonly used 2-D CNN, many scholars carry out ESC tasks from the perspective of 1-D CNN. Zhang et al.^12^ proposed an ESC method based on VGGNet^13^, and set the convolution filter to 1-D to learn the frequency and time characteristics of audio. Dai et al.^14^ proposed a 34 layer 1-D CNN model to classify the original one-dimensional waveform data, and showed a competitive accuracy with 2-D CNN based on Log-Mel spectrogram, but it needs a deeper convolution layer. Abdoli et al.^15^ proposed an end-to-end ESC method based on 1-D CNN, without artificial feature extraction. Antonio et al.^16^ proposed DENet, which used lossless original audio as input, and combined the proposed layer with a bidirectional gated recurrent unit to obtain a good audio classification effect. Francisco et al.^17^ developed the SinNet neural network architecture, which uses raw audio to classify animal sounds, and achieves rapid convergence in the case of limited data. Dong et al.^18^ proposed a Two-Stream convolutional neural network. The model is composed of 1-D CNN based on raw audio and 2-D CNN based on Log-Mel spectrogram. It combines the time and frequency characteristics of audio and achieves 95.7% average accuracy and 96.07% highest accuracy on UrbanSound8K.

In order to make ESC-related research better serve practical applications, based on this research, researchers have carried out research on the task of sound event localization and detection (SELD). Shimada et al.^19^ proposed a CRNN framework that combines CNN and RNN to realize the localization and detection of sound events, but the performance needs to be improved. Nguyen et al.^20^ replaced the backbone network in CRNN with VGG and ResNet, and proposed a new SALSA feature, which finally achieved excellent performance. And the author also tested the performance of the combination of the backbone network and different RNN structures. Sun et al.^21^ proposed Adaptive Hybrid Convolution based on the idea of matrix decomposition, and combined the attention module to obtain good results in the SLED task. Sudarsanam et al.^22^ replaced the RNN blocks in the CRNN architecture with self-attention blocks. They also investigate stacking multiple self-attention blocks, using multiple attention heads in each self-attention block, and position embedding and layer normalization. With the rise of Transformer research, this structure has also been applied to SELD. Huang et al.^23^ obtained performance no less than CRNN using the combination of CNN and Transformer.

In the above, we discuss a lot of ESC-related fields work based on CNN, but most of these works ignore one of the key issues in ESC tasks, that is real-time. Although Yousef et al.^24^ once proposed to construct a simple shallow model and a single MFCC feature for ESC, the essence is still to simply stack convolutional layers. Although the shallow CNN model improves the real-time classification to a certain extent, its lower model capacity makes it difficult to improve the classification accuracy.

In order to improve the operating efficiency of CNN models, many researches on CNN compression have been proposed successively. Li et al.^25^ proposed a neural network pruning method, which calculates the ${\mathcal{L}}_{1}$ norm of the elements in the filter as the saliency measure, and removes filters with small metric value to obtain a " thinner " network, reduce the running cost of the model, and finally make up for the loss of accuracy through fine-tuning. Further, Valerio et al.^26^ proposed a dynamic hard pruning method that progressively prunes low-contribution neurons during training, which not only reduces the size of the final neural network model, but also reduces the memory footprint during training, and accuracy loss due to the pruning operation is offset by a dynamic batch sizing method. Hinton et al.^27^ proposed the idea of knowledge distillation. Soft goals related to the teacher network are introduced to guide students network training, thereby realizing knowledge transfer. However, this method ignores the important structural knowledge of the teacher network. Later Tian et al.^28^ introduced contrastive learning into knowledge distillation to train student to capture significantly more information in the teacher’s representation of the data. Chen et al.^29^ proposed HashNet, which uses a hash function to group weights, and weights in the same hash bucket share the same value, thereby significantly reducing the model size. Dettmers^30^ used 8-bit approximation data type instead of 32-bit floating-point representation to improve the running speed of the model, and designed a dynamic tree data type to reduce approximation errors. For the purpose of extreme acceleration, the binarization network is also developed accordingly. Courbariaux et al.^31^ proposed BinaryConnect, which uses binarized weights during forward and backpropagation to train DNNs, but still maintains full-precision weights when computing gradients. Zhou et al.^32^ proposed Incremental Network Quantization (INQ), which transforms a full-precision network model into a lossless binarized version through iterative weight division, population quantization, and retraining, and can be accelerated by hardware shifting.

Most of the above CNN compression methods are carried out on the basis of the existing classical models, and the performance of the methods is affected by the baseline models. In addition, these models are mostly used in the field of computer vision. In order to better serve the ESC task, we proposed a lightweight model RACNN, which reduces the memory footprint of training and inference processes, and maximizes model performance within limited resources (storage and computing resources).

## Method

In this section, we introduce the proposed ESC method. First, we introduce the proposed RACNN model, and then we describe the preprocessing process of environmental sound data.

### Proposed RACNN Model

**Figure 2:**
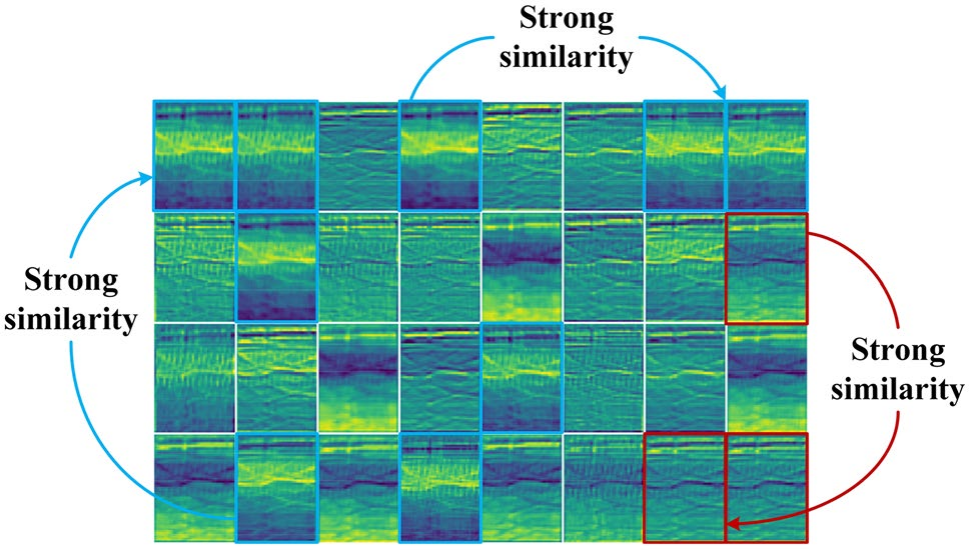
Display of feature maps for hidden middle layer output. (Heat maps are drawn by the plotting library Matplotlib 3.1.1. Detailed descriptions are available at https://matplotlib.org).

Deep convolutional neural network usually improves its accuracy by a large number of stacking convolution operations, such as AlexNet^7^, VGG^13^, ResNet^33^, which leads to a large amount of storage and computing resources consumption. However, we find that the feature maps output by the hidden middle layer of the complex model have great similarity. As shown in Fig. 2, these feature maps are obtained by the first layer of VGG-11 based on the Urbansound8K dataset. Feature maps marked with blue and black borders have strong similarity, which means that there is a lot of redundancy in convolution operation of CNN model. However, if the redundancy of middle feature maps in CNN model is reduced by simply scaling convolution channel, the accuracy will be reduced. Therefore, maintaining certain redundant feature maps plays a positive role in the final classification results.

In view of the strong similarity and high redundancy of feature maps output by the middle layer of the current mainstream CNN model, and the redundancy plays a positive role in the final classification results. We need to focus on reducing the resources required to generate these similar feature maps, that is, to find a cheap way to replace the filter used to generate these similar feature maps. For an intermediate convolution layer, given the input data $X \in {\mathbb{R}}^{c \times h \times w}$, where $c$ is the number of input data channels, $h$ and $w$ are the height and width of input data respectively. The $\ell^{th}$ convolution operation can be expressed as:

1 $$ Y = h\left( {\gamma_{\ell } \cdot norm\left( {x_{\ell - 1} *\theta_{\ell } + b_{\ell } } \right) + \beta_{\ell } } \right) $$

where * represents convolution operation, $\theta_{\ell } \in {\mathbb{R}}^{{c_{\ell - 1} \times c_{\ell } \times k_{h} \times k_{w} }}$ is the weight tensor, $k_{h}$ and $k_{w}$ is the height and width of the filter, $b_{\ell } \in {\mathbb{R}}^{{c_{\ell } }}$ is the bias term, $norm\left( x \right)$ is the batch normalization (BN) operation^34^, $\gamma_{\ell },\,\, \beta_{\ell } \in {\mathbb{R}}^{\ell }$ is the scale factor and the offset factor respectively, and $h\left( x \right)$ is the activation function. When convolution is performed, the parameter quantity and FLOPs can be obtained by the following formula:

2 $$ parameters = c_{\ell } \cdot \left( {c_{\ell - 1} \cdot k_{h} \cdot k_{w} + 1} \right) $$

3 $$ FLOPs = h \cdot w \cdot \left( {c_{\ell - 1} \cdot k_{h} \cdot k_{w} + 1} \right) \cdot c_{\ell } $$

At present, CNN generally uses convolution operation with high resolution and high channel number. The number of channels is 256 or 512, even thousands, so the parameters and FLOPs of convolution are huge.

#### Solution

**Figure 3:**
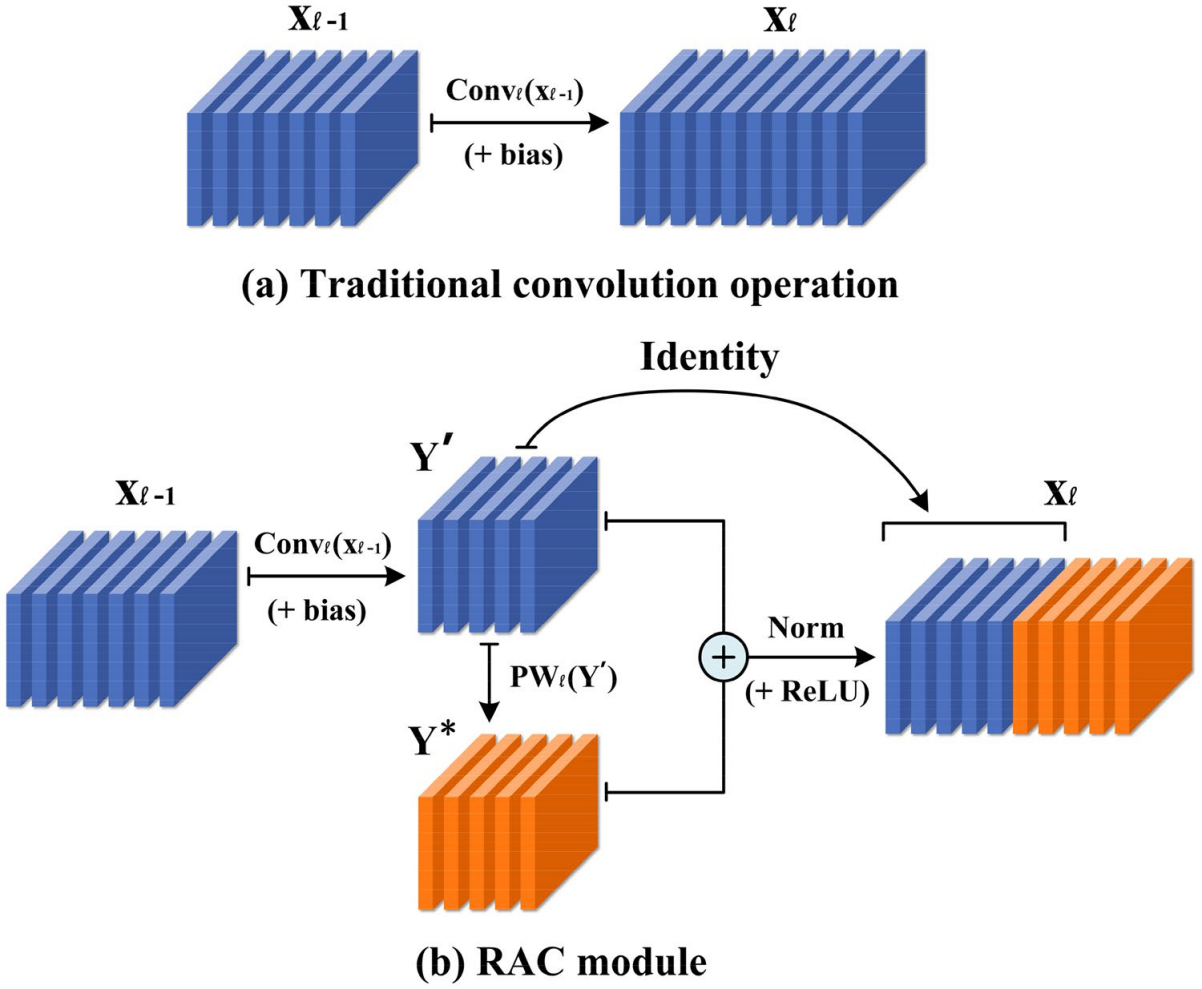
Example of traditional convolution operation and RAC module under the same number of output channels.

In view of the above analysis, this paper proposes the RAC module. As shown in Fig. 2, the feature maps output by the convolutional layer are very similar to each other. We believe that for these similar feature maps, we do not need to obtain them through expensive calculations. These similar feature maps are like replicas of inherent feature maps, which have limited performance improvements to the model, but consume a large number of parameters and FLOPs. Therefore, we can generate these redundant feature maps through a series of cheap operations on the basis of inherent feature maps. As shown in Fig. 3, first we generate the inherent feature maps $Y^{\prime} \in {\mathbb{R}}^{{h \times w \times c_{\ell }^{^{\prime}} }}$ through the conventional convolution operation:

4 $$ Y^{\prime} = X*\theta^{\prime}_{\ell } + b^{\prime}_{\ell } $$

where $\theta^{\prime}_{\ell } \in {\mathbb{R}}^{{c_{\ell - 1} \times c^{\prime}_{\ell } \times k_{h} \times k_{w} }}, \,\, b^{\prime}_{\ell } \in {\mathbb{R}}^{{c^{\prime}_{\ell } }}$ are the convolution parameters used to generate the inherent feature maps, and $c_{\ell }^{^{\prime}} < c_{\ell }$. Then take the inherent feature maps as input data, and generate the remaining feature maps $Y^{*} \in {\mathbb{R}}^{{h \times w \times \left( {c_{\ell } - c_{\ell }^{^{\prime}} } \right)}}$ through 1 × 1 pointwise convolution:

5 $$ Y^{*} = Y^{\prime}*\theta_{\ell }^{*} + b_{\ell }^{*} $$

where $\theta^{\prime}_{\ell } \in {\mathbb{R}}^{{c^{\prime}_{\ell } \times \left( {c_{\ell } - c^{\prime}_{\ell } } \right) \times 1 \times 1}} , b^{\prime}_{\ell } \in {\mathbb{R}}^{{c_{\ell } - c^{\prime}_{\ell } }}$, and combine it with the inherent feature maps merged. Enter it as input data to the next layer for processing:

6 $$ Y = Y^{\prime} \oplus Y^{*} $$

where $\oplus$ indicates that the connection is made on the channel. Compared with the commonly used 3 × 3, 5 × 5, and 7 × 7 convolution operations, the pointwise convolution can almost be ignored in the number of parameters and FLOPs. Moreover, each channel of the feature map obtained by pointwise convolution combines the information of all channels of the inherent feature maps, which makes the feature information contained in it more abundant. We can change the compression ratio by adjusting the ratio between the inherent feature maps and the feature maps generated by a cheap method.

#### Complexity analysis

The RAC module can generate the same number of feature maps as conventional convolutional layers with less resource consumption. Therefore, we can easily use the RAC module to upgrade the existing classical neural network architecture, thereby reducing the computational cost. Next, we will analyze in detail the effectiveness of the RAC module in reducing the number of parameters and FLOPs. We use the RAC module to replace the $\ell^{th}$ conventional convolution operation. Assuming that the ratio of the number of feature maps generated by pointwise convolution to the number of inherent feature maps is $\alpha$, then we can use Eq. (7) to calculate the parameter compression ratio of the RAC module compared with ordinary convolution:

7 $$ \begin{aligned} ratio_{p} &= \frac{{c_{\ell } \cdot \left( {c_{\ell - 1} \cdot k_{h} \cdot k_{w} + 1} \right)}}{{\left( {1 - \alpha } \right) \cdot c_{\ell } \cdot \left( {c_{\ell - 1} \cdot k_{h} \cdot k_{w} + 1} \right) + \alpha \cdot c_{\ell } \cdot \left[ {\left( {1 - \alpha } \right) \cdot c_{\ell } \cdot 1 \cdot 1 + 1} \right]}} \hfill \\ & = \frac{1}{{\left( {1 - \alpha } \right) + \frac{{\alpha \cdot \left[ {\left( {1 - \alpha } \right) \cdot c_{\ell } + 1} \right]}}{{c_{\ell - 1} \cdot k_{h} \cdot k_{w} + 1}}}} \approx \frac{1}{1 - \alpha } \hfill \\ \end{aligned} $$

Similarly, the acceleration ratio for FLOPs can be calculated by Eq. (3) to get $ratio_{F} \approx 1/\left( {1 - \alpha } \right)$. A trade-off between computational complexity and accuracy can be achieved by adjusting *α*. But when *α* = 1, the RAC module will degenerate into a regular convolution operation with a convolution kernel size of 1 × 1. However, 1 × 1 convolution will lead to performance degradation because it cannot capture the spatial relationship of feature information. Therefore, *α* should be reasonably valued according to the actual task.

#### Efficient network construction

**Figure 4:**
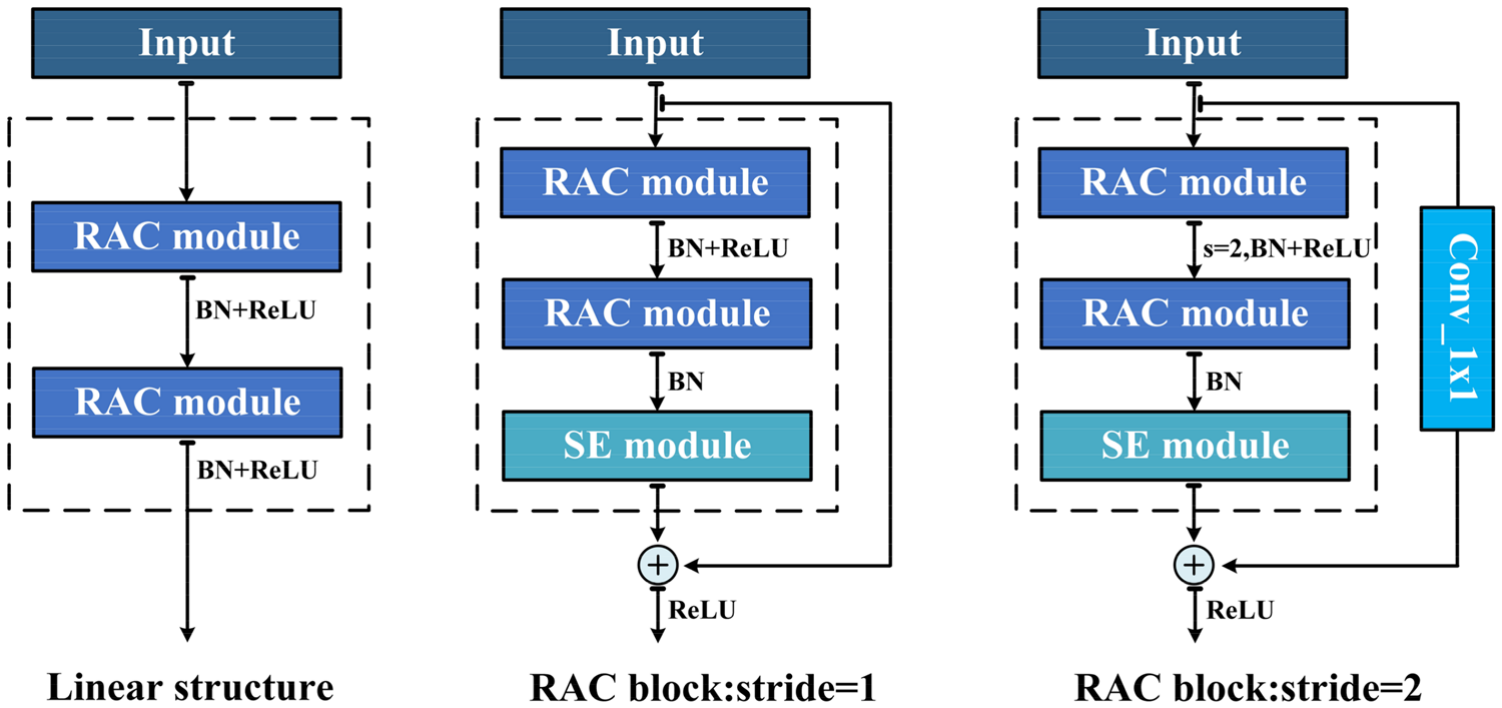
The illustration of RAC block.

Using the efficient RAC module and drawing on the idea of the residual module in ResNet, we designed the RAC block. As shown in Fig. 4, the RAC block integrates the RAC module, shortcut and the channel domain attention mechanism. The main part of the proposed RAC block is composed of two stacked RAC modules. After the first RAC module is over, we add BN^34^ and ReLU^35^ nonlinear activation layers. From ResNet's experience, only the BN layer is added after the second RAC module, and the ReLU nonlinear activation layer is added after the shortcut operation. The number of channels output by the two RAC modules can be adjusted according to specific needs. RAC block mainly has the following three structures: (a) Two RAC modules have the same output channel; (b) Compared with the second RAC module, the first RAC module has fewer output channels, so the first RAC module plays a role of dimensionality reduction. Through this structure, a more compact neural network can be obtained. We call this structure LRAC block; (c) More convolution channels are used in the first RAC module, which we call HRAC block. The first two structures are mainly used in our experiment. Practitioners can choose the most suitable structure according to their actual needs. After the second RAC module is over, we have selectively added the SE module^36^, by processing the obtained feature maps, a one-dimensional vector equal to the number of channels is obtained as the score of each channel, and then the score value is applied to the corresponding channel:

8 $$ \tilde{X}_{c} = w_{c} \cdot X_{c} $$

Finally, a shortcut connection is established between the input and output of the block:

9 $$ Y = F\left( {X,\theta } \right) + X $$

Among them, $F\left( {X,\theta } \right)$ represents the serial calculation of two RAC modules, and $\theta$ is the weight parameter of the calculation. When the number of channels of the input data and output data of the block is not uniform, we perform dimensionality increase and dimensionality reduction operations through pointwise convolution to achieve shortcut connections. If the stride of the RAC block is 2, the pointwise convolution with stride of 2 is also used to complete the down-sampling operation.

**Figure 5:**
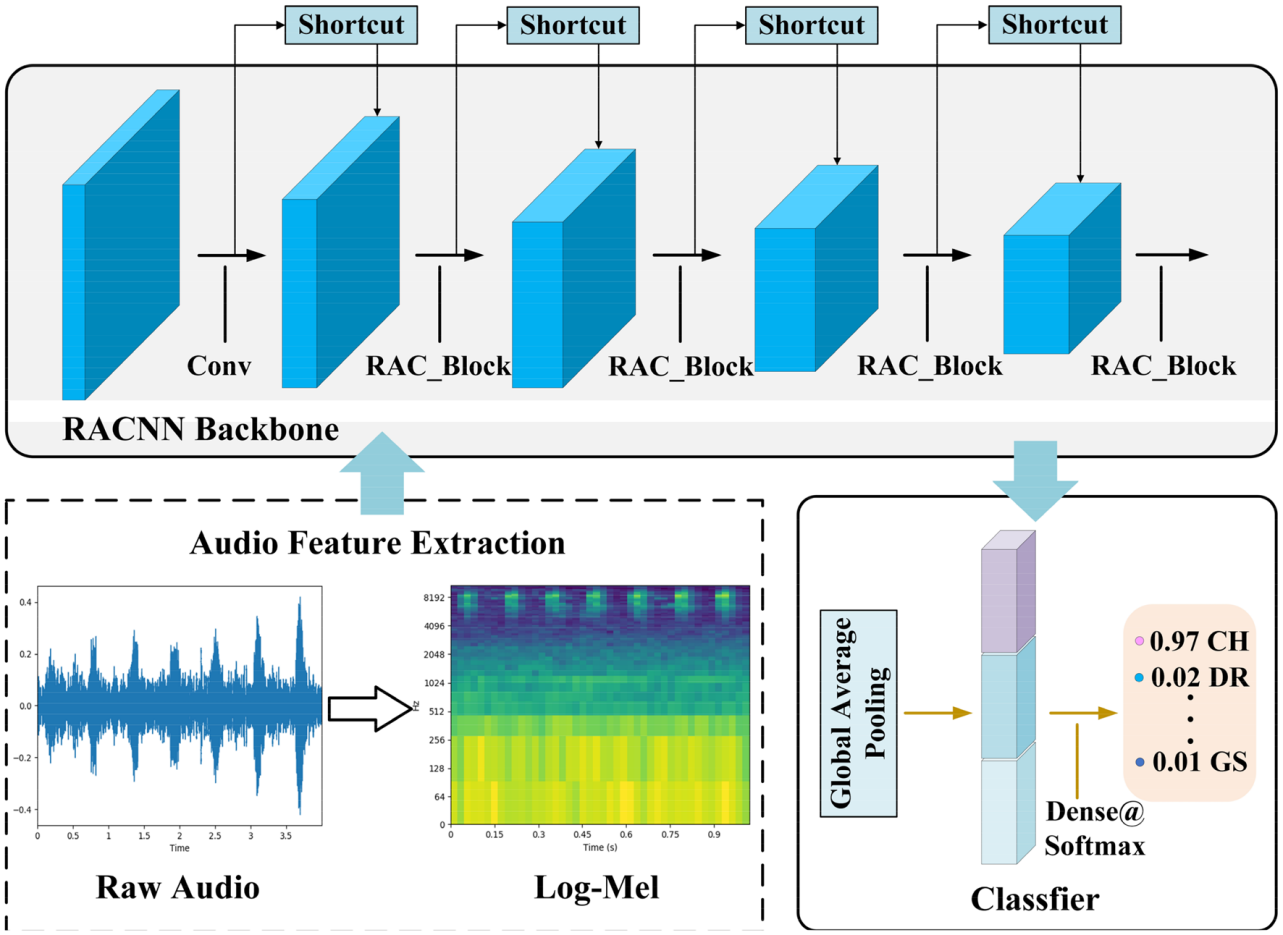
The forward propagation process of the RACNN model.

On the basis of RAC block, RACNN is formed by simple stacking. As shown in Table 1, we follow the advantages of ResNet's basic architecture. For the input samples, we first perform a 3 × 3 convolution operation to extract features and improve the dimension of features. Followed by the RAC block with 16, 32, 64, 128 output channels in turn, and a down-sampling operation of a multiple of 2 is performed as the number of channels increases. Next is the global average pooling (GAP) layer, through which the feature map is turned into a one-dimensional vector, and finally a dense connection layer accompanied by the softmax function is added to complete the classification operation. The dropout operation is also applied to some layers of RACNN. The specific forward propagation process is shown in Fig. 5. The proposed architecture only provides a basic reference. Further tuning of hyperparameters or exploration of the architecture will further improve the performance of RACNN.

**Table 1:** The overall architecture of RACNN. “exp” represents the scaling ratio of the number of output channels of the first RAC module in the LRAC block.

Input	Operator	Output	exp	Stride	SE block
60 × 44 × 1	Conv2d_3 × 3	60 × 44 × 16	–	(1,1)	–
60 × 44 × 16	RAC_Block	60 × 44 × 16	–	(1,1)	$\surd$
60 × 44 × 16	RAC_Block	30 × 22 × 32	–	(2,2)	$\surd$
30 × 22 × 32	LRAC_Block	15 × 11 × 64	0.75	(2,2)	$\surd$
15 × 11 × 64	LRAC_Block	8 × 6 × 128	0.75	(2,2)	$\surd$
8 × 6 × 128	GAP	128	–	–	–
1 × 1 × 128	Dense	10	–	–	–

For different scenarios in reality, we can use a smaller model to achieve faster resolution or a larger model to achieve higher classification accuracy on specific tasks. We can simply multiply the output channel of each layer by a coefficient *μ* uniformly, and change the width of the neural network through this coefficient. By adjusting the width coefficient *μ*, we can easily trade-off between delay and performance.

#### Different from existing methods

(a) Different from the widely used depthwise convolution, the RAC module can fuse the feature information of multiple channels, fully learn the spatial information of the feature maps, and improve the performance of classification. (b) Different from the Inception series model. Although different kernel sizes are also used in the Inception module at the same time, different from our serial structure, the Inception module uses a parallel structure. This method has the following disadvantages: First, the convolution with different kernel sizes in parallel structure accepts all the feature channels, while the point convolution of the RAC module only accepts some channels. Therefore, in terms of parameters and FLOPs, our method needs Lower than the Inception structure. Secondly, the conclusion drawn from the research in^37^, the operating efficiency of the serial structure is higher than that of the parallel structure, so the RAC module has lower latency. (c) We show through experiments that, in terms of accuracy, the serial structure of the RAC module is also better than the parallel structure used by the Inception module.

### Data preprocessing

#### Feature extraction

The experimental process involves four datasets: UrbanSound8K^38^, ESC-10, ESC-50^39^ and TAU-NIGENS Spatial Sound Events 2021 development dataset^40^. Different from speech recognition, environmental sound event (ESE) usually contains more noise, so it is more difficult to recognize. The mel filterbank is closer to imitating the response of the human auditory system. Because the human ear’s perception of sound is not linear, it is better described by the non-linear relationship of log. Therefore, Log-Mel is often used to process voice data. Relying on Log-Mel features for neural network training.

#### Zero-padding

**Figure 6:**
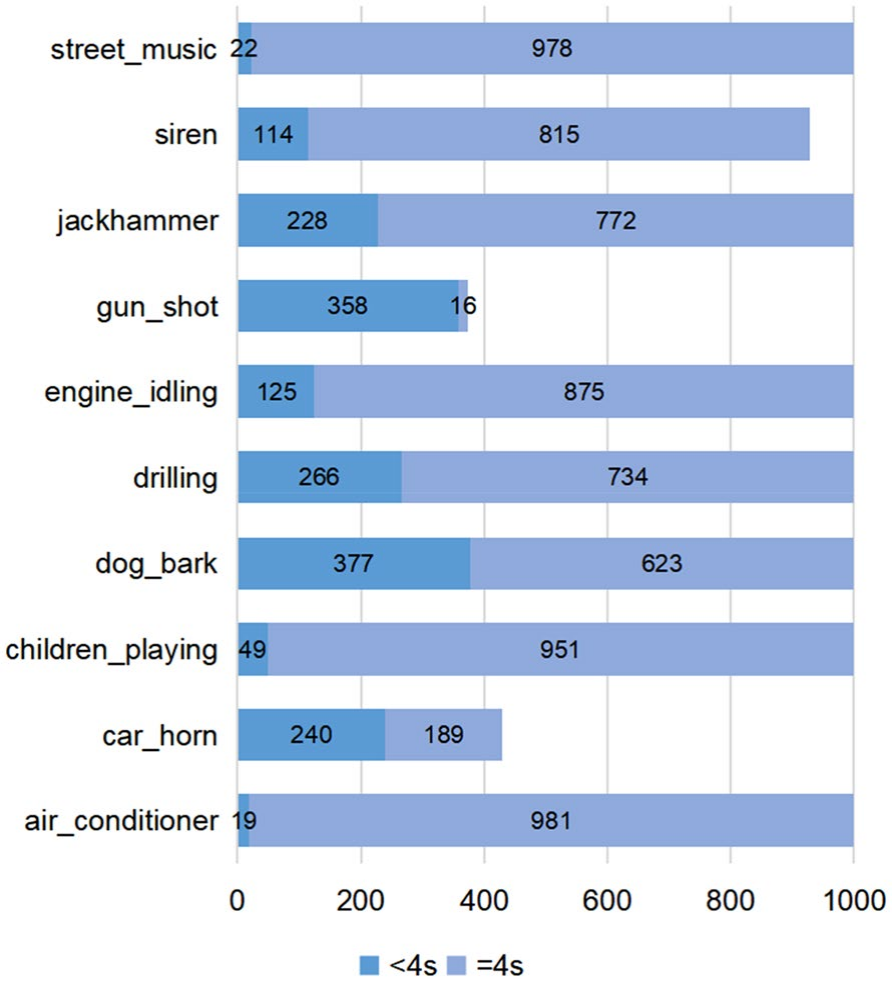
UrbanSound8K dataset (Dark blue represents sample < 4 s, light blue represents sample > 4 s).

**Figure 7:**
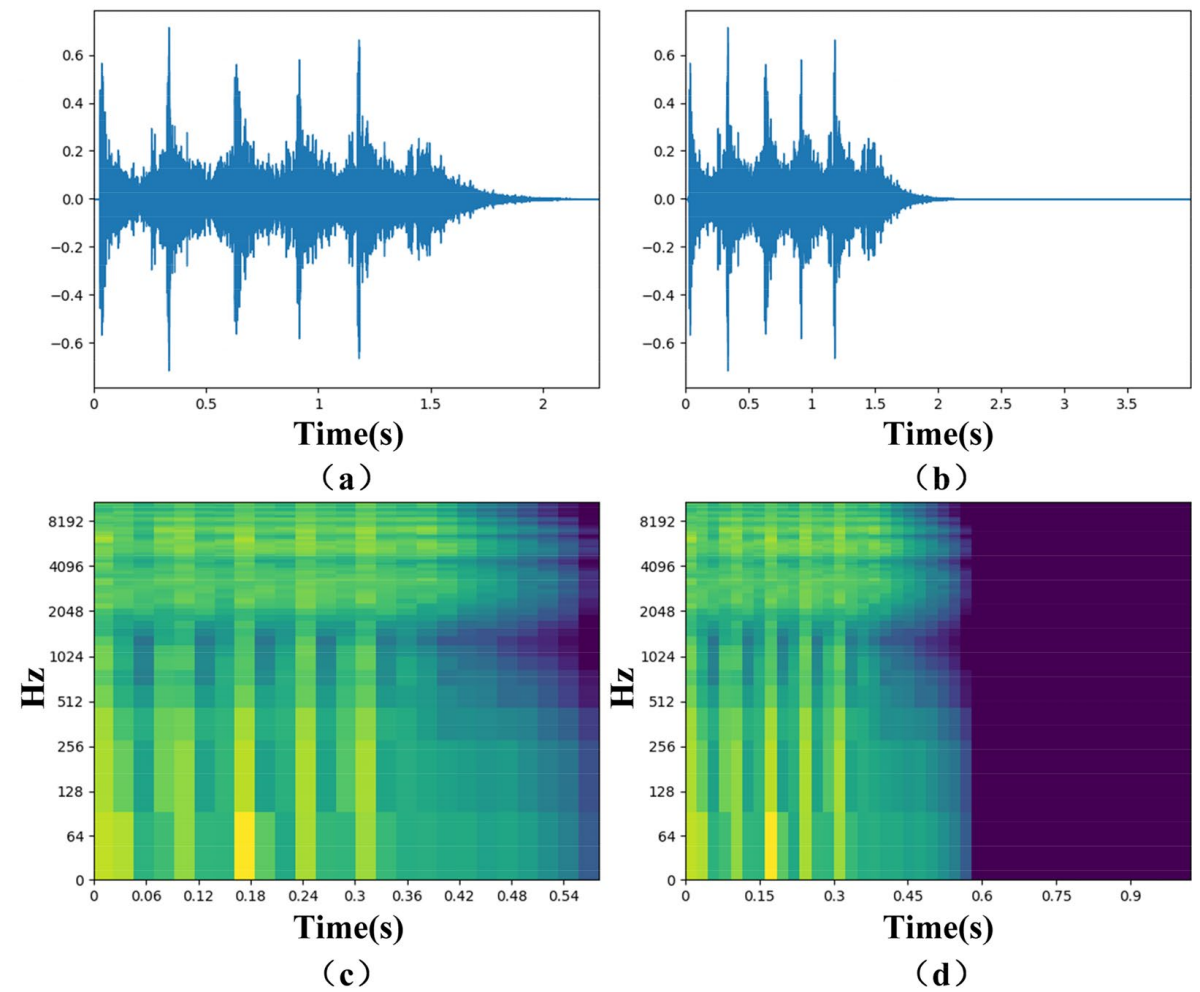
Visual display of audio data before and after zero padding. ((**a**) and (**b**) are drawn by the plotting library Matplotlib 3.1.1, (**c**) and (**d**) are drawn by the audio processing library Librosa 0.8.1).

As a public dataset, UrbanSound8K is often used in ESC related research. This dataset contains 10 categories and a total of 8732 samples (≤ 4 s). Among them, there are 1798 less than 4 s, as shown in Fig. 6. However, neural networks usually require fixed-size inputs. If such data samples are discarded, it will cause serious waste of dataset. And for samples of categories such as gun shots, most of the samples are less than 4 s. If only samples with a duration equal to 4 s are used for training, it is very easy to cause over-fitting and reduce model performance. In addition, the length of samples collected in real scenarios is usually difficult to be unified. To this end, we use zero padding method for data repair, that is, for data samples whose duration is less than 4 s, we directly fill in by zero padding. Although this method is very simple, it has shown good performance in the experiment. This method keeps about 20% of the data while ensuring the duration of the data samples is consistent. As shown in Fig. 7, (a) and (b) are the original data before and after zero padding, and (d) and (e) are their corresponding Log-Mel spectrogram.

#### Data augmentation

The ESC-50 data set has a small number of data samples (2000 data in total, 40 in each category), so it is easy to cause over-fitting. We performed data enhancement operations on the audio data to enhance the generalization ability of the model. We mainly performed the following operations on audio data:

- Pitch shift augmentation. By scaling the frequency to adjust the pitch, we increase and decrease the audio data signal to a certain extent. Here we set the amplitude factor to + 2/−2.
- Time shift augmentation. The scale changes in the time dimension, and the audio data is stretched or accelerated. In this paper, we stretch the sound clip to 1.2 times its original length, and then cut it to its original length.

In summary, the specific ESC framework proposed in this paper is shown in Algorithm1.
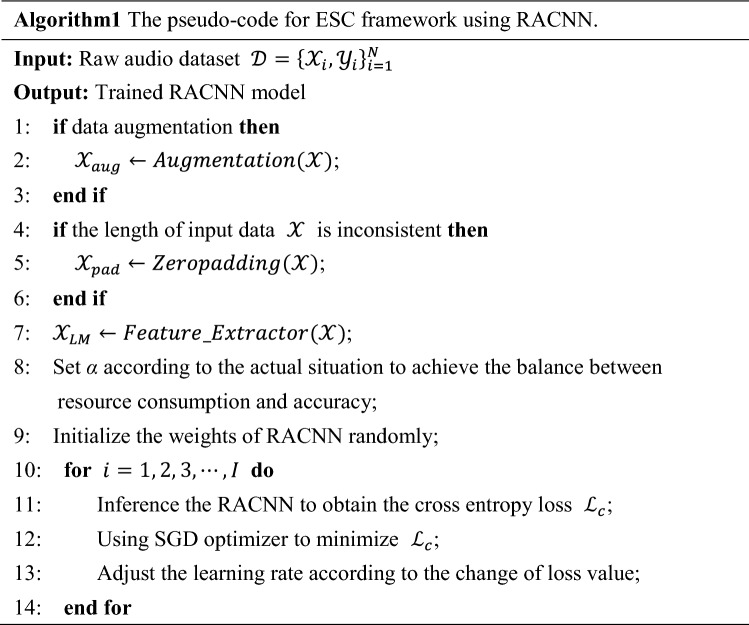


## Experiment

### ESC Dataset

The UrbansSound8K dataset contains 10 sound categories, namely air condition (AC), car horn (CH), children playing (CP), dog barking (DB), drilling (DR), engine idling (EI), gunshots (GS) , jackhammer (JA), siren (SI) and street music (SM). A total of 8732 samples, with a total duration of 9.7 h, are unevenly distributed in each category. In view of the non-uniform data sample length, most of the data is 4 s, so zero padding method is used to fill the samples with less than 4 s to 4 s. The ESC-10 dataset also contains 10 categories, namely dog (DG), rooster (RO), rain (RA), sea waves (SW), crackling (CR), crying baby (CB), sneezing (SN), clock tick (CT), helicopter (HE), chainsaw (CS). The ESC-50 dataset collects 5 major classes including animals, natural soundscapes, water sounds, human non-verbal sounds, interior/domestic sounds, exterior/urban noises, a total of 50 categories. Because of the large number of categories, we use No. 1–50 said. The dataset includes a total of 2000 samples, the samples in each category are evenly distributed, each audio sample is 5 s in length, and a total of 2.8 h. ESC-10 is a subset of 10 classes (400 samples in total) selected from ESC-50.

### SELD dataset

The TAU-NIGENS Spatial Sound Events 2021 development dataset comes from the DCASE2021 challenge, and it has two types of data, one is the microphone array (MIC) and the other is the first-order amisonic (FOA). We use the FOA format for experiments. The development dataset consists of 600 one-minute audios with a sampling frequency of 24 kHz and is divided into 400, 100 and 100 for training, validation and testing respectively.

### Feature extraction

For the above UrbanSound8K and ESC-10 datasets, use the Librosa audio processing package to read the raw data with a sampling rate of 11025 Hz, the number of channels of the Log-Mel spectrogram is 60, the frame shift is 1024, and the size of the finally extracted Log-Mel matrix for 60 × 44. For ESC-50, we set the number of channels to 128 and the frame shift to 431 to obtain a Log-Mel matrix with a size of 128 × 128. For the TAU-NIGENS Spatial Sound Events 2021 development dataset, this paper adopts the same feature extraction method as in^21^.

### Hyperparameter settings

We use a stochastic gradient descent (SGD) optimizer with a multi-step learning rate strategy to train the proposed model. The momentum weight of the Nesterov momentum we use is 0.9 without damping, and a weight decay of 5 × 10^–4^. Batch size is set to 32. For the ESC datasets, the initial learning rate is set to 0.1. The model on UrbanSound8K is trained for 120 epochs, the learning rate is multiplied by the attenuation coefficient 0.1 every 40 epochs, and the final result is obtained using tenfold cross-validation. The models on ESC-10 and ESC-50 are trained for 300 epochs, the learning rate is multiplied by the attenuation coefficient 0.1 every 100 epochs, and the final result is obtained using fivefold cross-validation. For the SELD dataset, an initial learning rate of 0.001 was used and a decay factor of 0.1 was multiplied every 100 epochs until the loss on the validation set no longer decreased. We take the model that performs the best on the validation set and report its performance on the test set. Finally, we report "mean ± variance".

### Compare with parallel structure

**Figure 8:**
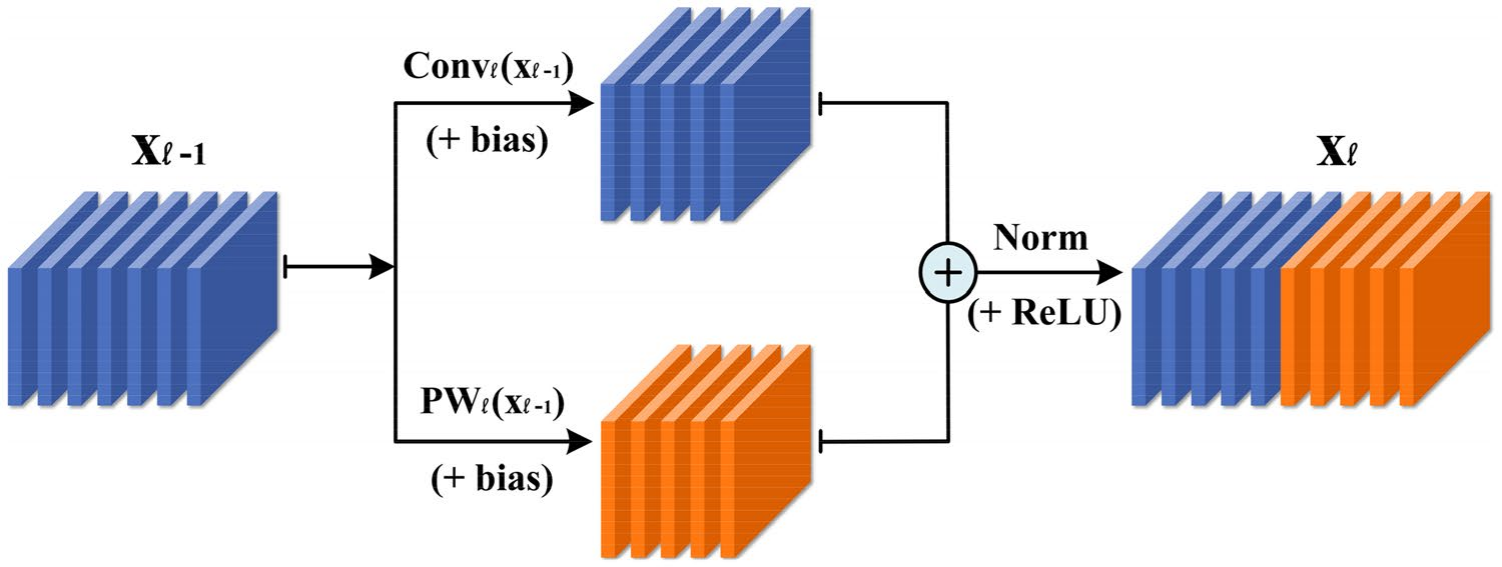
The parallel structure of the RAC module.

**Figure 9:**
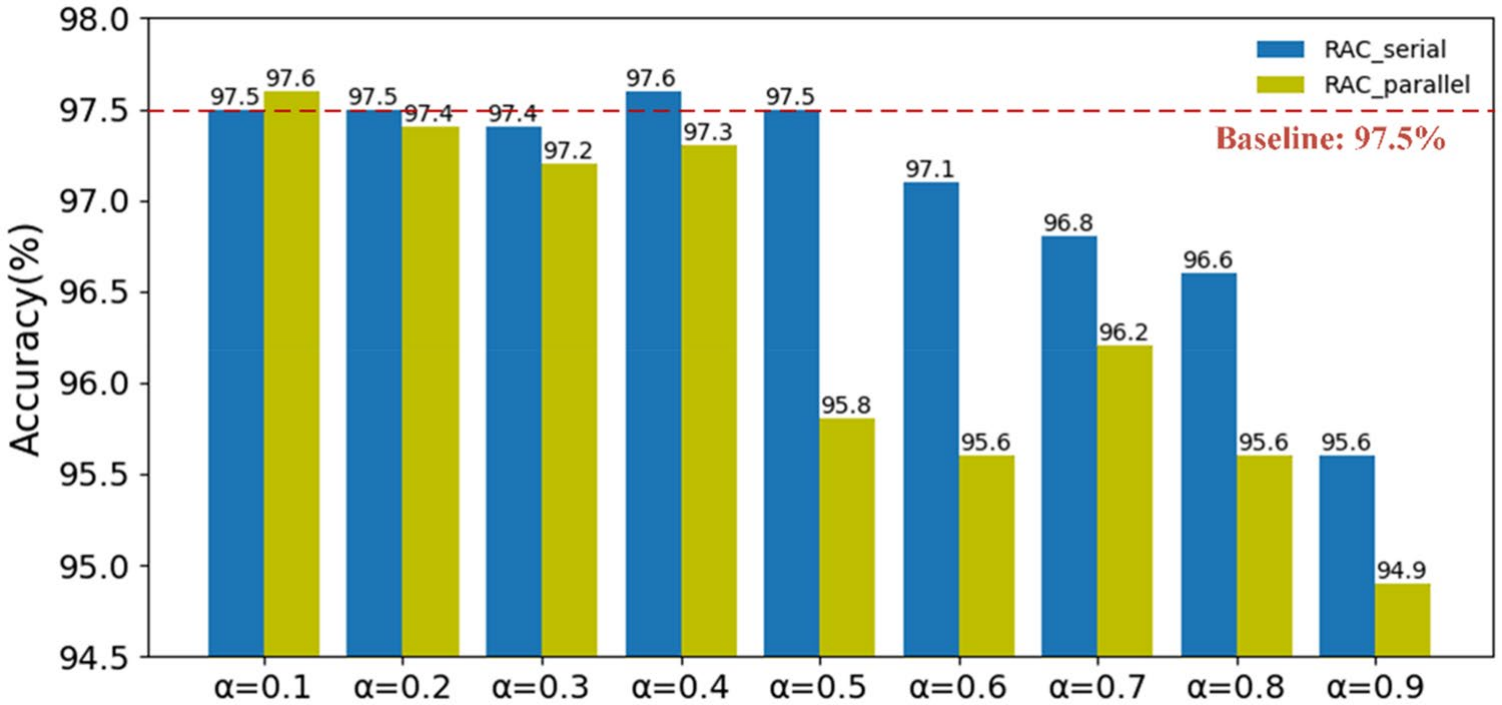
The performance of RAC module under different structures.

The research in Ma et al.^37^ has shown that the parallel structure is not conducive to the improvement of computing efficiency. We further test the performance of the two structures on UrbanSound8K. The parallel structure of the RAC module is shown in Fig. 8. We test the performance of the two structures under different compression ratios by adjusting the ratio *α* of pointwise convolution to the original convolution. The details are shown in Fig. 9. The data in the figure represent the accuracy. Under different ratio *α*, the accuracy of the RAC module of the serial structure is almost better than that of the RAC module of the parallel structure, in which “Baseline” represents the RACNN model using traditional convolution operation. Therefore, it can be concluded that the RAC module we proposed not only has higher computing efficiency, but also performs well in performance.

### Results on the UrbanSound8K dataset

**Figure 10:**
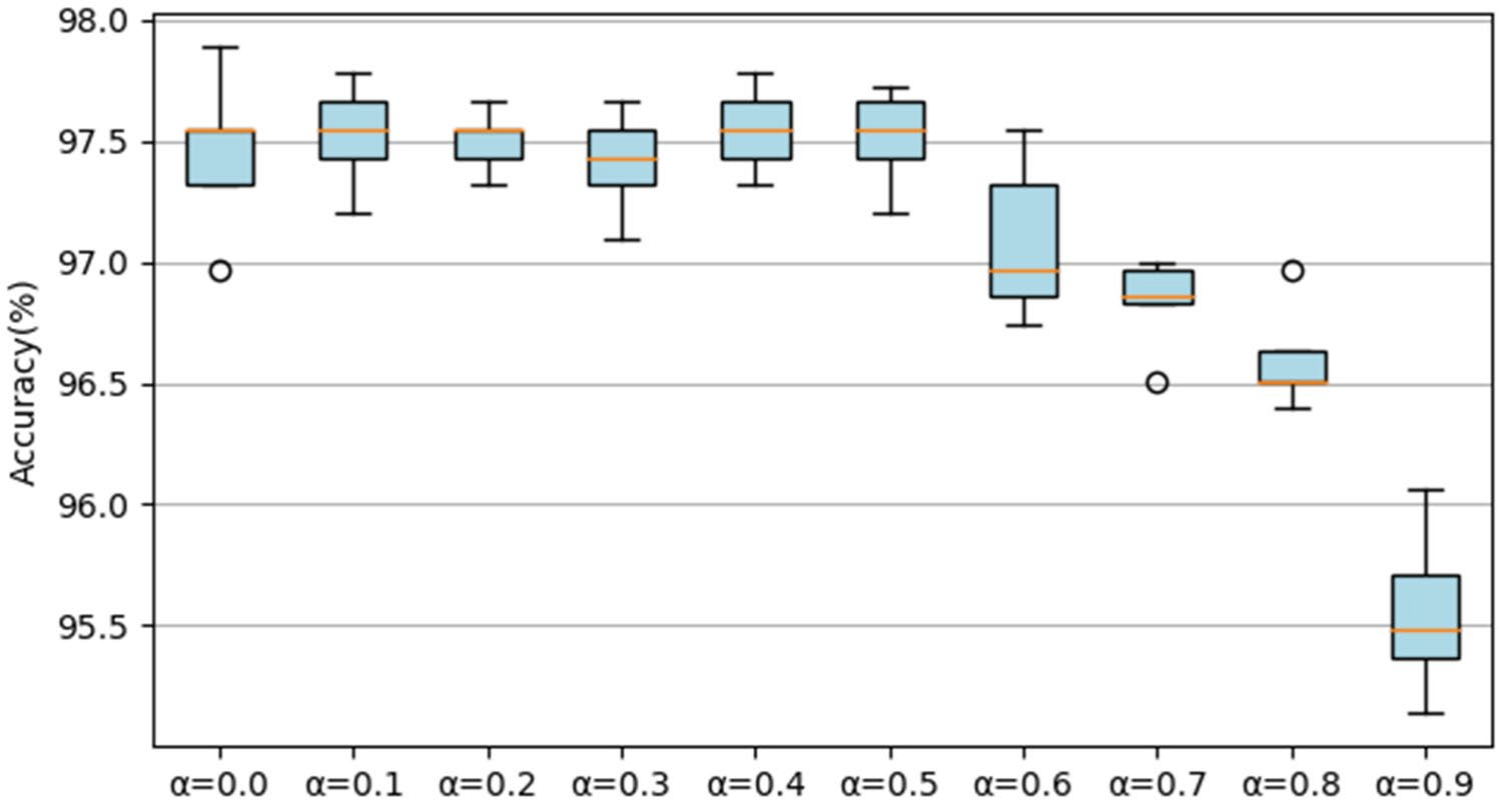
The accuracy of the RACNN model on the UrbanSound8K dataset under different *α*.

**Figure 11:**
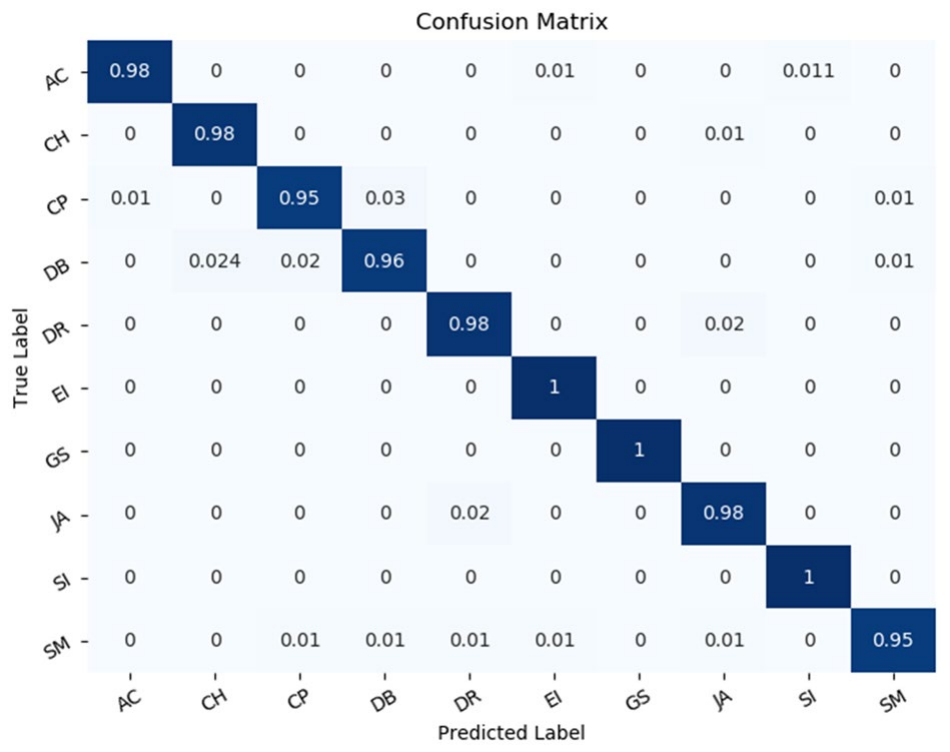
Confusion Matrix of RACNN on UrbanSound8K dataset.

First, we test the accuracy of RACNN under different ratios of *α*. A s shown in Fig. 10, when *α* ≤ 0.5, as the parameters and FLOPs decrease, the accuracy of the model is relatively stable. When *α* > 0.5, the accuracy of the model shows a rapid decline. When *α* = 1, the accuracy is reduced to 85.07% (± 0.64%) (not shown in the Fig. 10), and the accuracy fluctuates greatly. Therefore, keeping a certain number of 3 × 3 convolutions is beneficial to the final result. Based on comprehensive considerations, we select the model obtained by *α* = 0.5 as our final model for classifying the UrbanSound8K dataset. When *α* is set to 0.5, not only the overall classification result is high (97.51% (± 0.18%)), the classification result of each class is also outstanding. The confusion matrix is shown in Fig. 11. Except children's playing and street music sounds, the recognition accuracy of other types of sounds exceeded 95%, and the classification accuracy of the three sounds of engine idling, gunshot, and siren even reached 100%. Subsequently, the parameters and FLOPs of the model under different proportions of *α* are reported, as shown in Table 2, by adjusting the value of *α*, the accuracy and efficiency can be flexibly balanced.

**Table 2:** The number of parameters and FLOPs of RACNN on UrbanSound8K under different *α*.

Layer	*α* = 0.0		*α* = 0.1		*α* = 0.3		*α* = 0.5		*α* = 0.7	
	Param	FLOPs	Param	FLOPs	Param	FLOPs	Param	FLOPs	Param	FLOPs
Conv2d	160	422.4 K	160	422.4 K	160	422.4 K	160	422.4 K	160	422.4 K
RAC_Block	4.8 K	12.2 M	4.5 K	11.6 M	3.7 M	9.5 M	2.6 K	6.5 M	1.7 K	4.2 M
RAC_Block	15.0 K	9.5 M	13.9 K	8.8 M	11.5 K	7.2 M	8.7 K	3.8 M	5.9 K	3.5 M
LRAC_Block	45.8 K	7.2 M	42.6 K	6.7 M	34.9 K	5.4 M	26.7 K	4.1 M	18.7 K	2.7 M
LRAC_Block	182.8 K	8.4 M	169.4 K	7.7 M	139.1 K	6.3 M	106.0 K	4.7 M	72.7 K	3.1 M
Dense_10	1.3 K	1.3 K	1.3 K	1.3 K	1.3 K	1.3 K	1.3 K	1.3 K	1.3 K	1.3 K
Total	251.6 K	37.8 M	231.9 K	35.2 M	190.8 K	28.8 M	145.7 K	21.0 M	100.5 K	13.9 M

### Results on the ESC-10 dataset

For the ESC-10 dataset, since the dataset is simpler than the UrbanSound8K dataset, we simplified the RACNN used on the UrbanSound8k dataset. As shown in Table 3, for each RAC block, we multiply the number of output channels by 0.5, while for the first convolutional layer of RACNN, we did not do any processing. We found that reducing the number of output channels of the first convolutional layer will seriously reduce the accuracy of the model. Therefore, it is necessary to ensure a certain number of convolution channels to fully extract the features of the input data, otherwise it will cause the loss of feature information and affect the performance of the model.

**Table 3:** The number of parameters and FLOPs of RACNN on ESC-10 under different *α*.

Layer	*α* = 0.0		*α* = 0.2		*α* = 0.3		*α* = 0.4		*α* = 0.5	
	Param	FLOPs	Param	FLOPs	Param	FLOPs	Param	FLOPs	Param	FLOPs
Conv2d	160	422.4 K	160	422.4 K	160	422.4 K	160	422.4 K	160	422.4 K
RAC_Block × 0.5	1.9 K	5.0 M	1.7 K	4.4 M	1.5 K	3.9 M	1.3 K	3.3 M	1.1 K	2.8 M
RAC_Block × 0.5	3.8 K	2.4 M	3.2 K	2.0 M	3.0 K	1.9 M	2.6 K	1.6 M	2.2 K	1.3 M
LRAC_Block × 0.5	11.5 K	1.8 M	9.9 K	1.5 M	8.9 K	1.4 M	8.0 K	1.2 M	6.7 K	1.0 M
LRAC_Block × 0.5	45.8 K	2.1 M	39.0 K	1.8 M	34.9 K	1.6 M	31.1 K	1.4 M	26.7 K	1.2 M
Dense_10	650	650	650	650	650	650	650	650	650	650 K
Total	64.8 K	11.7 M	54.6 K	10.2 M	49.0 K	9.1 M	43.8 K	8.0 M	37.5 K	6.7 M

**Figure 12:**
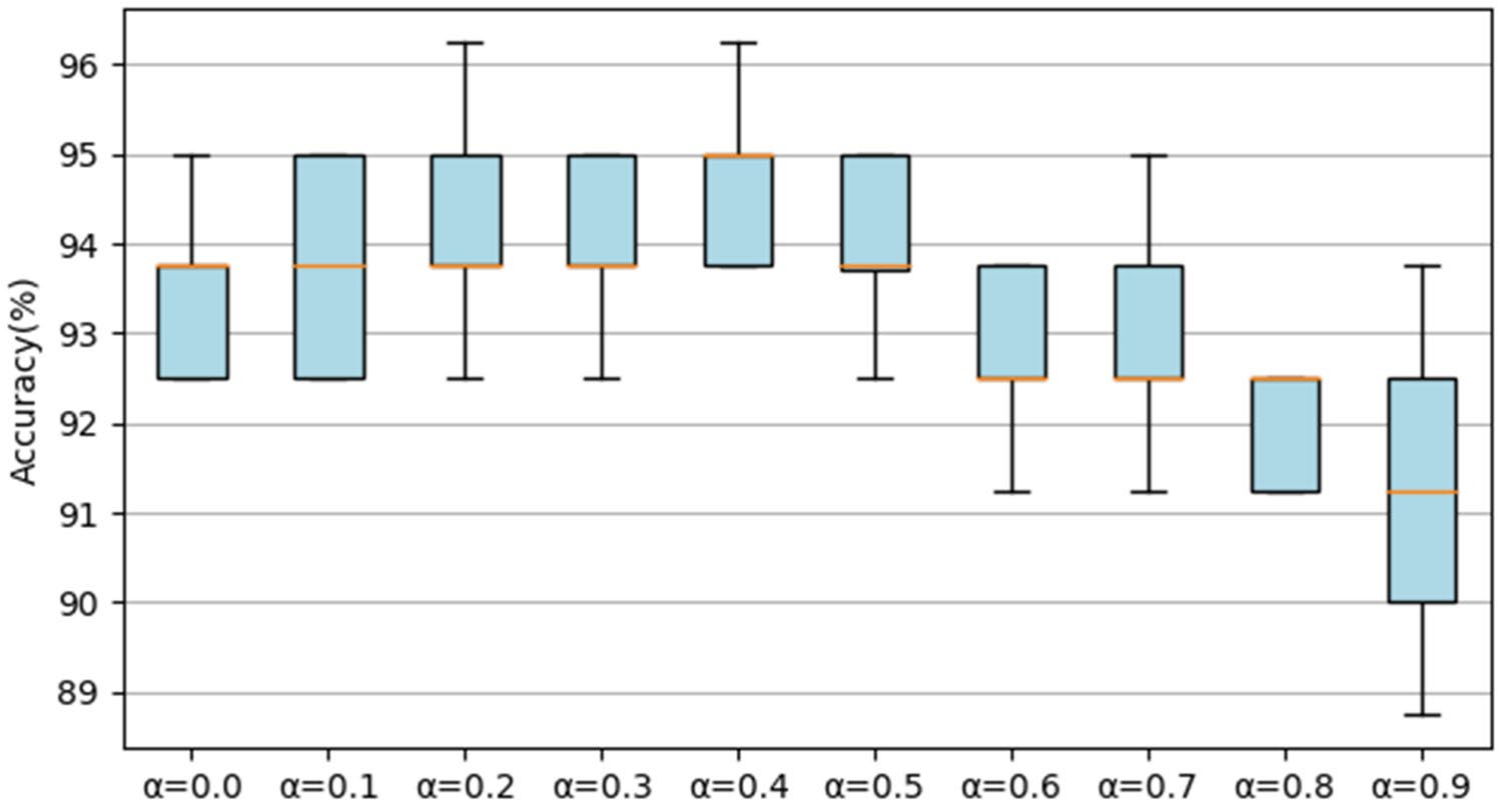
The accuracy of the RACNN model on the ESC-10 dataset under different *α*.

**Figure 13:**
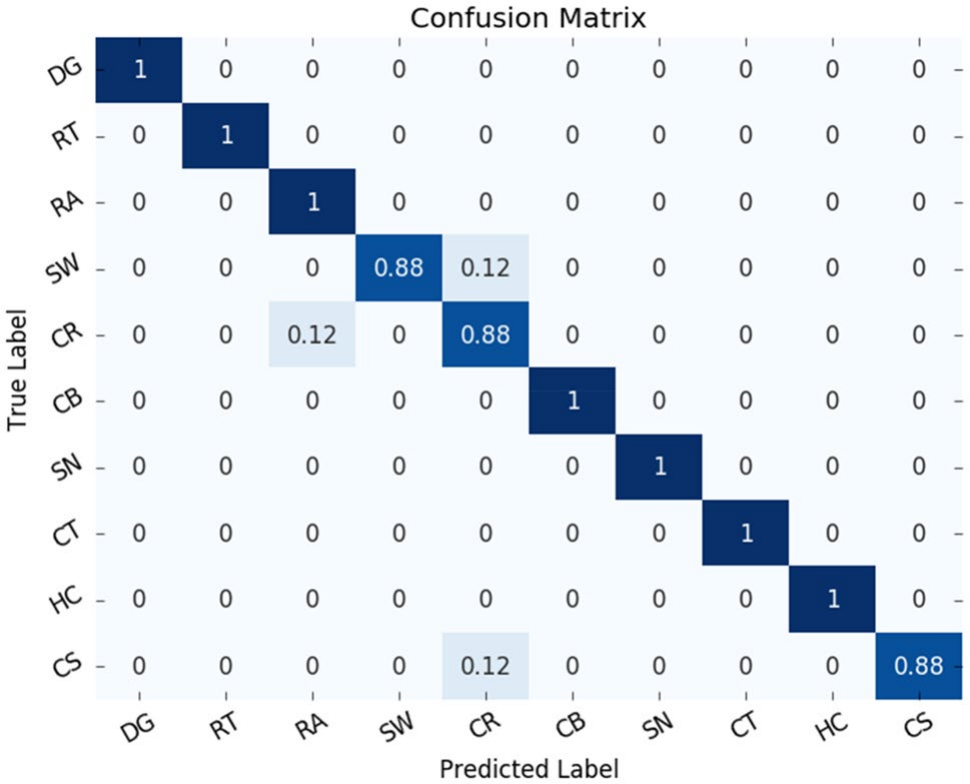
Confusion Matrix of RACNN on ESC-10 dataset.

We also conduct experiments by changing the value of *α* and the resource consumption under different *α* is reported in Table 3. As shown in Fig. 12, when *α* ≤ 0.5, as the model consumes less resources, the performance of the model does not fluctuate significantly. The performance is best when *α* = 0.4. And when *α* = 1, similar to RACNN on UrbanSound8K, there will be a significant decrease in accuracy (67.50% (± 1.76%)) and huge fluctuations. For the selected final model, we not only reported the overall accuracy rate (94.75% (± 0.93%)), but also reported the accuracy rate in different categories. The specific confusion matrix is shown in Fig. 13. RACNN has reached 100% accuracy on the seven sounds of dog, rooster, rain, crying baby, sneezing, clock trick, and helicopter. The accuracy of sea waves, cracking, and chainsaw is also acceptable (88%).

### Results on the ESC-50 dataset

In order to make the experiment closer to real application scenarios and verify the performance of the RACNN model in real scenarios, we conduct experiments on the ESC-50 dataset. The ESC-50 dataset is more complex than the above two datasets, with more classification categories and less training data, so it is very easy to overfit during the training process. For this reason, the width of RACNN has been doubled as a whole, and the larger model capacity enables it to have stronger feature processing capabilities. At the same time, we expand the input feature matrix to 128 × 128. Although this approach will increase FLOPs to a certain extent, the richer feature information significantly improves the classification effect. During the experiment, we found that the use of the SE module did not increase the performance of the RACNN on the ESC-50. For this reason, in the experiment on ESC-50, we removed the SE module in the RAC block.

**Figure 14:**
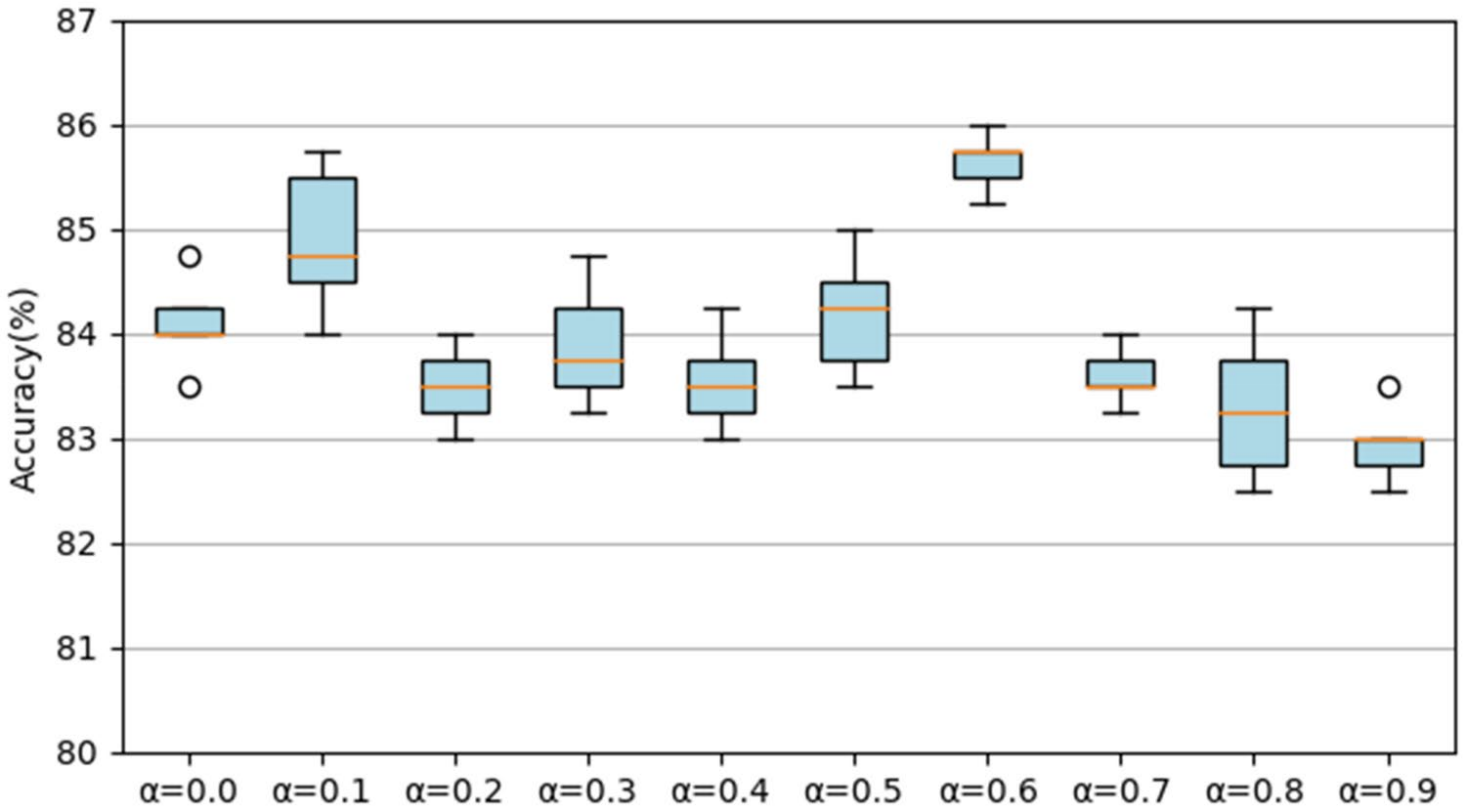
The accuracy of the RACNN model on the ESC-50 dataset under different *α*.

**Figure 15:**
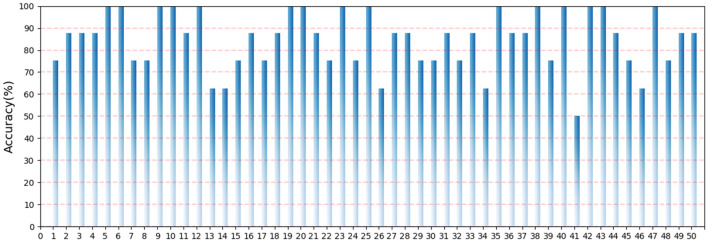
Classification results of RACNN model on the ESC-50 dataset.

In order to find a suitable *α* to achieve a balance between accuracy and efficiency, we compared the accuracy of RACNN models under different *α* and the resource consumption under different *α* is reported in Table 4. As shown in Fig. 14, when *α* = 0.6, the model obtains the best classification accuracy (86.65% (± 0.25%)). When a = 1, the model cannot converge on the test set due to the inability to capture the spatial connection of the time–frequency feature information. In addition, when *α* = 0.2, 0.3 and 0.4, the performance also decreased relatively. The reason for the analysis is that the amount of training data is small and the classification granularity is fine, so the over-parameterization of the model leads to the phenomenon of over-fitting. At the same time, we also tested the performance of the model in different categories. Because the ESC-50 dataset contains a large number of categories, we did not show the confusion matrix, but showed the accuracy of 50 categories in the form of a histogram. As shown in Fig. 15, except for No. 41, other categories have reached acceptable accuracy, and even 15 categories have reached an accuracy of 100%.

**Table 4:** The number of parameters and FLOPs of RACNN on ESC-50 under different *α*.

Layer	*α* = 0.0		*α* = 0.1		*α* = 0.3		*α* = 0.6		*α* = 0.8	
	Param	FLOPs	Param	FLOPs	Param	FLOPs	Param	FLOPs	Param	FLOPs
Conv2d	320	5.2 M	320	5.2 M	320	5.2 M	320	5.2 M	320	5.2 M
RAC_Block × 2	18.5 K	303.0 M	16.9 K	277.6 M	13.7 K	224.9 M	8.0 K	131.8 M	4.4 K	72.8 M
RAC_Block × 2	55.4 K	227.0 M	50.9 K	208.6 M	40.7 K	166.8 M	24.6 K	100.6 M	12.7 K	52.0 M
RAC_Block × 2	221.4 K	226.8 M	203.5 K	208.4 M	162.6 K	166.5 M	98.0 K	100.4 M	50.5 K	51.7 M
RAC_Block × 2	885.2 K	226.6 M	810.4 K	207.5 M	649.9 K	166.4 M	388.0 K	99.3 M	201.4 K	51.6 M
Dense_10	12.9 K	12.9 K	12.9 K	12.9 K	12.9 K	12.9 K	12.9 K	12.9 K	12.9 K	12.9 K
Total	1.2 M	988.7 M	1.1 M	907.3 M	880.2 K	729.8 M	531.8 K	437.4 M	282.2 K	233.3 M

### Comparison with the state-of-the-art methods

The final results are shown in Table 1. We compare the proposed method with the state-of-the-art method on UrbanSound8K, ESC-10 and ESC-50.

#### UrbanSound8K

For this dataset, we compare the proposed model with several state-of-the-art models in the ESC field such as M18^15^, Piczak-CNN^4^, EnvNet-v2^41^, Pro-CNN^42^, Pyramid-Combined CNN^44^, Two-Stream^18^, TCNN-DS^10^, etc. It can be seen from Table 5 that compared to M18^14^, Piczak-CNN^4^ and EnvNet-v2^41^, we have obtained absolute improvements of 25.81%, 23.81%. and 19.21%, respectively. Pro-CNN^42^, Su-CNN^11^, Two-Stream^18^, TCNN-DS^10^ have also obtained good accuracy, but these models all use the feature fusion method, while RACNN only uses a single Log-Mel spectrogram feature. Despite this, the RACNN model still maintains an advantage in accuracy. Practitioners can choose appropriate sound features to input into RACNN. Since this is not the focus of our work, we have not discussed this in detail. In addition to accuracy, another important evaluation index of ours is the number of parameters and FLOPs, which is also the focus of our work. Since most of the work does not report its FLOPs, and FLOPs are affected by the size of the input data. Therefore, we do not make relevant comparisons in Table 5. We only report on the FLOPs of RACNN in Tables 3 and 4. From the data in the table, we can see that the FLOPs of RACNN are acceptable. We compare RACNN with the state-of-the-art models in detail in terms of the number of parameters. Our RACNN model on UrbanSoun8K only contains 145.7 K trainable parameters, which is only about 7% of the smallest Two-Stream^18^ (2.1 M). With excellent performance and fewer parameters and FLOPs, the RACNN model can be more effectively deployed on resource-constrained embedded devices and perform more real-time classification.

**Table 5:** Comparison of RACNN and state-of-the-art methods on UrbanSound8K dataset.

Method	Feature	UrbanSound8K	
		Acc(%)	Param
M18^14^	Raw data	71.7	3.7 M
Piczak-CNN^4^	Log-Mel	73.7	26 M
EnvNet-v2^41^	Raw data	78.3	18 M
Pro-CNN^42^	Log-Mel + Raw data	91.9(± 0.5)	–
Su-CNN^11^	MFCC + Log-Mel + CST	93.4	6.6 M
DCNN^43^	Log-Mel	94.14	3.17 M
Pyramid-Combined CNN^44^	Spectrogram	94.8	–
Two-Stream^18^	Log-Mel + Raw data	95.8	2.1 M
TCNN-DS^10^	Multiple Features	97.2	12.9 M
RACNN	Log-Mel	97.51(± 0.18)	145.7 K

#### ESC-10

Due to the small number of data samples in this dataset, we make subtle adjustments to the RACNN on ESC-10, and make the structure more compact by scaling the model. At the same time, compared with several state-of-the-art models such as SoundNet^45^, ACRNN^46^, Multi-Stream CNN^47^, WaveMsNet^48^, etc. The results are shown in Table 6. RACNN still achieves the best performance when only a single Log-Mel spectrogram feature is used. It is proved that the proposed RAC block has high computational efficiency and strong feature extraction ability, and can fully extract the key information in the input data for the next classification operation. In terms of parameters, the RACNN on ESC-10 only contains 43.8 K of trainable parameters. Compared with the WaveMsNet model (1.38 M), it probably achieves a compression of 31.5× .

**Table 6:** Comparison of RACNN and state-of-the-art methods on ESC-10 and ESC-50 dataset.

Method	Feature	ESC-10		ESC-50	
		Acc(%)	Param	Acc(%)	Param
Pyramid-Combined CNN^44^	Spectrogram	78.14	–	81.4	–
Piczak-CNN^4^	Log-Mel	80.5	26 M	65.0	26 M
DCNN^43^	Log-Mel	81.25	3.17 M	57	3.17 M
Two-Stream^18^	Log-Mel + Raw data	87.25	2.1 M	–	–
EnvNet-v2^41^	Raw data	91.4(± 0.1)	18 M	81.6	18 M
SoundNet^45^	Raw data	92.1	–	74.2	–
Pro-CNN^42^	Log-Mel + Raw data	92.1(± 0.6)	–	82.8	–
ACRNN^46^	Log Gammatone	93.7	3.81 M	86.1	3.81 M
Multi-Stream CNN^47^	Wav + STFT + delta	93.7	–	83.5	–
WaveMsNet^48^	Multi-scale features	93.75(± 0.63)	1.38 M	79.1	13.8 M
RACNN	Log-Mel	94.75(± 0.93)	43.8 K	85.65(± 0.25)	531.8 K

#### ESC-50

This dataset is extremely challenging. Its larger number of categories, finer-grained categories and limited trainable data make it difficult for neural network models to fit its data features to achieve high-precision classification. As shown in Table 6, the RACNN model has reached an accuracy rate that competes with state-of-the-art methods (the average accuracy rate is 85.65%, the highest accuracy rate is 86%), and the number of parameters of the RACNN model is still at a minimum level. Because we only use the Log-Mel spectrogram as the input of the model, the FLOPs of the RACNN model are at a low level.

### Sound event localization and detection

In order to verify the generalization of RACNN, we apply RACNN to the task of sound event localization and detection (SELD). SELD is composed of two subtasks, sound event detection (SED) and direction-of-arrival estimation (DOAE), so it is more challenging. CRNN^19^ has become the mainstream method in SELD field since it was proposed. Therefore, based on this framework, we combine RACNN to localize and detect sound events. CRNN is mainly composed of three parts: backbone convolutional layers, recurrent layers and transcription layers. The audio data is extracted from the feature sequence by the backbone network and sent to the bidirectional gate recurrent unit (BiGRU) of the recurrent layer for context information learning, and finally the output of the BiGRU is input to the two parallel branches of the fully connected block of the transcription layer to complete the sound event location and detection. We verify the performance of RACNN on SELD by replacing the backbone convolutional network in CRNN with RACNN and training the model using the ACCDOA^49^ output format. The specific RACNN structure is shown in Table 7.

**Table 7:** The overview of RACNN used in SELD.

Input	Operator	Output	exp	Stride	SE block
300 × 64 × 7	Conv2d_3 × 3	300 × 64 × 64	–	(1,1)	–
300 × 64 × 64	MaxPooling	60 × 64 × 64	–	(5,1)	–
60 × 64 × 64	HRAC_Block	60 × 64 × 64	1.5	(1,1)	$\surd$
60 × 64 × 64	MaxPooling	60 × 32 × 64	–	(1,2)	–
60 × 32 × 64	HRAC_Block	60 × 32 × 64	1.5	(1,1)	$\surd$
60 × 32 × 64	MaxPooling	60 × 16 × 64	–	(1,2)	–
60 × 16 × 64	HRAC_Block	60 × 16 × 64	1.5	(1,1)	$\surd$
60 × 16 × 64	MaxPooling	60 × 8 × 64	–	(1,2)	–
60 × 8 × 64	HRAC_Block	60 × 8 × 64	1.5	(1,1)	$\surd$
60 × 8 × 64	MaxPooling	60 × 4 × 64	–	(1,2)	–
60 × 4 × 64	HRAC_Block	60 × 4 × 64	1.5	(1,1)	$\surd$
60 × 4 × 64	MaxPooling	60 × 2 × 64	–	(1,2)	–

We compare RACNN with other lightweight models and model compression methods. In Table 7^21^, using the idea of matrix decomposition to build a lightweight model for SELD^25^. means using model pruning to compress ResNet14. MobileNet-V1 and MobileNet-V2 have also adapted the convolutional channels according to this task. To evaluate the performance of SELD, the official evaluation metrics^52^ from the DCASE2021 challenge are introduced in the experiments. As shown in Table 8, RACNN still achieves better performance with similar or even lower number of parameters and floating-point operations. In addition, in order to further verify the effectiveness of the method, we obtained the Uniform model by directly scaling the number of channels of RACNN, but the Uniform did not achieve the desired effect. It shows that maintaining a certain number of feature maps is beneficial to the model, so it is a correct direction to obtain a lightweight model by reducing the generation cost of feature maps.

**Table 8:** The performance comparison for different methods on the SELD dataset.

Method	Param	FLOPs	Test set			
			$\downarrow$ ER_20°_	$\uparrow$ F_20°_(%)	$\downarrow$ LE_CD_	$\uparrow$ LR_CD_ (%)
Baseline	0.5 M	123 M	0.69	33.9	24.1	43.9
Sun^21^	1 M	–	0.57	52.6	19.6	58.1
${\mathcal{L}}_{1}$-ResNet14-40%^25^	2.4 M	560 M	0.568	52.82	19.60	58.57
${\mathcal{L}}_{1}$-ResNet14-50%^25^	2.2 M	484 M	0.571	52.74	19.74	59.12
MoblieNet-V1^50^	1 M	468 M	0.586	51.64	20.61	57.64
MobileNet-V2^51^	0.80 M	490 M	0.583	51.92	20.44	57.76
Uniform × 0.7	0.75 M	525 M	0.573	52.53	19.74	58.13
Uniform × 0.5	0.58 M	346 M	0.592	52.14	20.75	57.61
RACNN(*α* = 0.0)	0.98 M	902 M	0.554	53.34	19.37	59.11
RACNN(*α* = 0.3)	0.83 M	675 M	0.556	53.29	19.38	58.97
RACNN(*α* = 0.5)	0.72 M	497 M	0.561	53.50	19.45	58.93

## Conclusion

In this paper, we propose a lightweight resource adaptive convolutional neural network (RACNN). After observing the feature maps output by the hidden middle layer, we found that there are similarities between many feature maps. We consider lower resource consumption to obtain these redundant feature maps. Based on this, we propose the RAC module. It can obtain the same number of feature maps as traditional convolution operations through less resource consumption, and adjust resource consumption according to actual needs. Although the RAC module can simply upgrade the existing CNN, in order to better extract abstract features for classification operations, we propose an efficient feature extraction block-RAC block based on the RAC module, and build RACNN by simply stacking RAC block. We first conduct experiments on the UrbanSound8K, ESC-10 and ESC-50 datasets. Compared with state-of-the-art models, the RACNN model not only maintains a leading position in accuracy, but the number of parameters and FLOPs of the RACNN model are much lower than these models. This makes the proposed RACNN model easier to transplant to embedded devices that lack storage and computing resources, and has more real-time processing capabilities. We also use RACNN for SELD task, demonstrating it’s excellent generalization performance. In the work of this article, we only use a single feature of the Log-Mel spectrogram. In future work, we will evaluate the performance of different features and mixed features on RACNN, so as to give full play to the performance of RACNN and improve the generality of the model. In addition, we will also consider fusing RACNN with the current mainstream CNN compression methods to further reduce its running cost and improve inference speed.
